# An upper limit for macromolecular crowding effects

**DOI:** 10.1186/2046-1682-4-13

**Published:** 2011-05-31

**Authors:** Andrew C Miklos, Conggang Li, Courtney D Sorrell, L Andrew Lyon, Gary J Pielak

**Affiliations:** 1 Department of Chemistry, University of North Carolina, Chapel Hill, North Carolina 27599, USA; 2 State Key Laboratory of Magnetic Resonance and Molecular and Atomic Physics, Wuhan Institute of Physics and Mathematics, Chinese Academy of Sciences, Wuhan, 430071, PR China; 3 School of Chemistry & Biochemistry, Georgia Institute of Technology, Atlanta, GA 30332, USA; 4 Petit Institute for Bioengineering and Bioscience, Georgia Institute of Technology, Atlanta, GA 30332, USA; 5 Department of Chemistry, University of Alberta, Edmonton, AB, T6G 2G2, Canada; 6 Department of Biochemistry and Biophysics, University of North Carolina, Chapel Hill, North Carolina 27599, USA; 7 Lineberger Comprehensive Cancer Center, University of North Carolina, Chapel Hill, North Carolina 27599, USA

## Abstract

**Background:**

Solutions containing high macromolecule concentrations are predicted to affect a number of protein properties compared to those properties in dilute solution. In cells, these macromolecular crowders have a large range of sizes and can occupy 30% or more of the available volume. We chose to study the stability and ps-ns internal dynamics of a globular protein whose radius is ~2 nm when crowded by a synthetic microgel composed of poly(*N*-isopropylacrylamide-*co*-acrylic acid) with particle radii of ~300 nm.

**Results:**

Our studies revealed no change in protein rotational or ps-ns backbone dynamics and only mild (~0.5 kcal/mol at 37°C, pH 5.4) stabilization at a volume occupancy of 70%, which approaches the occupancy of closely packing spheres. The lack of change in rotational dynamics indicates the absence of strong crowder-protein interactions.

**Conclusions:**

Our observations are explained by the large size discrepancy between the protein and crowders and by the internal structure of the microgels, which provide interstitial spaces and internal pores where the protein can exist in a dilute solution-like environment. In summary, microgels that interact weakly with proteins do not strongly influence protein dynamics or stability because these large microgels constitute an upper size limit on crowding effects.

## Background

**Figure 1:**
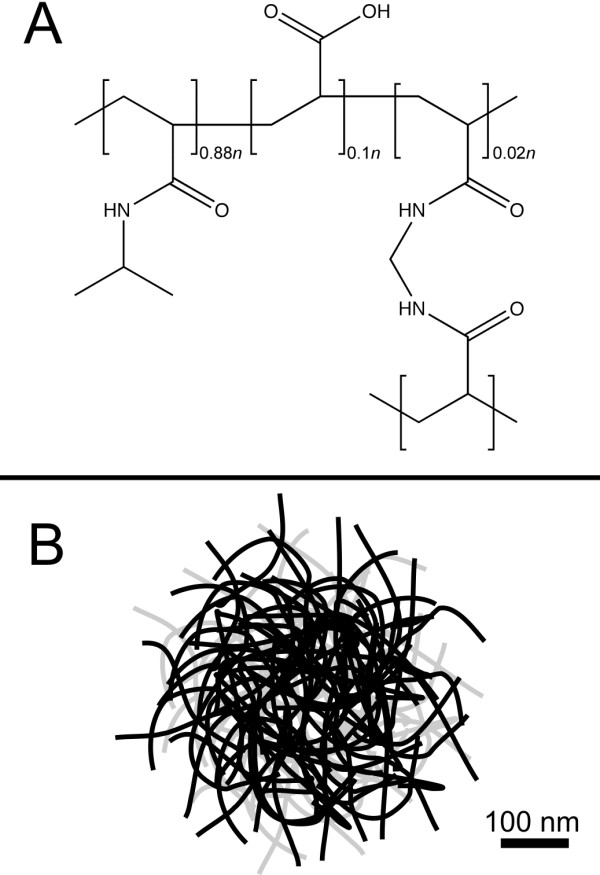
**Structure and Size of *p*-NIPAm-*co*-AAc**: A) The monomeric repeat of NIPAm. B) Overall shape and size of *p*-NIPAm-*co*-AAc microgels.

The cellular interior, where most biological processes occur, is unlike the dilute solutions where most proteins are studied. The large volume excluded by high macromolecule concentrations in cells, from 20-40% [1], is predicted to change many protein properties compared to dilute solution. We used a synthetic microgel composed of poly(*N*-isopropylacrylamide-*co*-acrylic acid) [*p*-NIPAm-*co*-AAc (Figure 1A)], as a crowding agent to study the backbone dynamics and the stability of the globular test protein, chymotrypsin inhibitor 2 (CI2).

*p*-NIPAm-*co*-AAc is of interest in pharmaceutical applications because it forms environmentally sensitive microgels [2]. Each microgel particle (Figure 1B) is a lightly cross-linked single polymer molecule of molecular weight 10^9 ^Da with an average of 70 monomer units between each cross link. The polymer absorbs a large amount of water resulting in spherical particles of 300 nm radii that exclude large amounts of solution volume. Their porosity arises from the balance between the external (solution) osmotic pressure and the internal osmotic pressure. This internal pressure is the result of the solvated cations that neutralize the deprotonated polymer side chains. We chose this crowding agent because its status as a drug delivery molecule makes it pharmaceutically relevant, and its ability to take up water provides a model for volume exclusion by a molecule much larger than our test protein.

CI2 is a small globular protein (7.4 kDa, PDB ID: 2CI2) that exhibits two-state folding [3]. NMR relaxation experiments [4] allowed us to assess backbone rotational dynamics for CI2 in the presence and absence of *p*-NIPAm-*co*-AAc. Amide proton exchange experiments [5,6] allowed us to assess the stability of CI2 in dilute and crowded conditions.

Globular proteins are often treated like hard spheres, but they have measurable amounts of internal motion. Analysis of relaxation parameters from NMR experiments - longitudinal and transverse relaxation times, *T*_*1 *_and *T*_*2 *_, and the ^15^N-^1^H nuclear Overhauser effect (NOE) of backbone ^15^N atoms - offers a residue-level window into this ps-ns backbone motion. The analysis involves a model-free method established by Lipari and Szabo [7]. Analysis is performed by fitting the spectral density function *I*(*ω*) as calculated from measured *T*_*1*_, *T*_*2*_, and NOE values [8], to the equation [7]

The overall correlation time *τ *is linked to the correlation time for isotropic tumbling, *τ*_*m*_, and internal motion timescale, *τ*_*e*_, by the equation

with the internal motions faster than the overall isotropic tumbling. The order parameter, *S*^*2*^, can have values between 0 and 1, and is related to the degree of internal mobility for a particular ^1^H-^15^N vector. An *S*^*2 *^value of 0 corresponds to complete freedom of motion. In this instance, relaxation is related solely to internal motion. An *S*^*2 *^value of 1 corresponds to complete restriction of the vector with respect to overall molecule motion, and relaxation is related solely to isotropic tumbling of the protein. These parameters can be linked to models for motion, in our case, the "wobble-in-a-cone" model [7]. Variations of Lipari-Szabo analysis exist for cases involving ms timescale conformational exchange, but no CI2 residue (except Thr40) has significant contributions from slow exchange [9]. It is also possible to study the equilibrium thermodynamic stability of globular proteins by using NMR.

Amide proton exchange experiments can be used with NMR to assess protein stability. The technique relies on the exchange of amide protons for deuterons in a D_2_O solution. We have recently reviewed the requirements for its application in crowded solution by using NMR [10].

Exchange occurs *via *the scheme

with opening rate *k*_*op*_, closing rate *k*_*cl*_, and rate of exchange from the open state *k*_*int*_. If the protein is stable (*k*_*cl *_> >*k*_*op*_) and exchange from the open state is rate limiting, the stability of an amide proton against exchange () can be determined with the equation,

where *R *is the gas constant and *T *is the absolute temperature. The value of *k*_*obs*_, the overall rate of exchange for any particular backbone amide proton, is assessed by acquiring ^1^H-^15^N heteronuclear single quantum correlation (HSQC) spectra as a function of time after initiating exchange. As with dynamics,  can be quantified on a per-residue basis. The largest  values match the global protein stability values determined by other methods (*e.g.*, calorimetry, circular dichroism spectropolarimetry) [11].

## Results

Experiments were performed by using samples comprising 1 mM CI2 in 50 mM sodium acetate solution, pH 5.4 at 37°C. Crowded samples also contained 10 g/L *p*-NIPAm-*co*-AAc microgels.

### Polymer Characterization

The microgels composed of *p*-NIPAm-*co*-AAc have an average hydrodynamic radius (*R*_*H*_) of 312 nm and an average polydispersity of 7.4%. The molecular weight of the microgels was estimated to be 1 GDa by multiple angle laser light scattering [12].

### Controls for Amide Proton Exchange

To determine whether exchange from the open state (*k*_*int*_) is rate limiting, nuclear Overhauser enchancement spectroscopy-detected amide proton exchange (NOESY-HEX) experiments were performed [10]. The results are given in Table 1, along with individual backbone residue decay rates from HSQC-detected amide proton exchange.

**Table 1:** NOESY-HEX results

Residue(s)	k_obs _NOESY (s^-1 ^× 10^5^)	k_obs _HSQC (s^-1 ^× 10^5^)
Leu8	3	3
Val9	2	2
Leu8 + Val9^a^	5	5
Leu8, Val9^b^	5	N/A
Lys17	52	40
Lys18	20	15
Lys17 + Lys18^a^	72	55
Lys17, Lys18^b^	50	N/A
		
Ala58	3	4
Glu59	3	3
Ala58 + Glu59^a^	6	7
Ala58, Glu59^b^	7	N/A

*k*_*abs *_values from NOESY-detected amide proton exchange and HSQC-detected amide proton exchange for CI2 in 10 g/L *p*-NIPAm-*co*-AAc, 50 mM sodium acetate, pH 5.4, 37°C.

^a ^Sum of values from individual crosspeak decays

^b ^Exchange rate of amide-amide NOESY crosspeak

To determine whether *k*_*int *_values are changed by crowding, phase-modulated clean exchange (CLEANEX-PM) experiments [13] were used to determine *k*_*int *_for residues on the extended loop region of CI2. For His37, *k*_*int *_values were 11 ± 2 s^-1 ^in dilute solution and 8 ± 2 s^-1 ^in 10 g/L *p*-NIPAm-*co*-AAc.

### Dynamics

**Figure 2:**
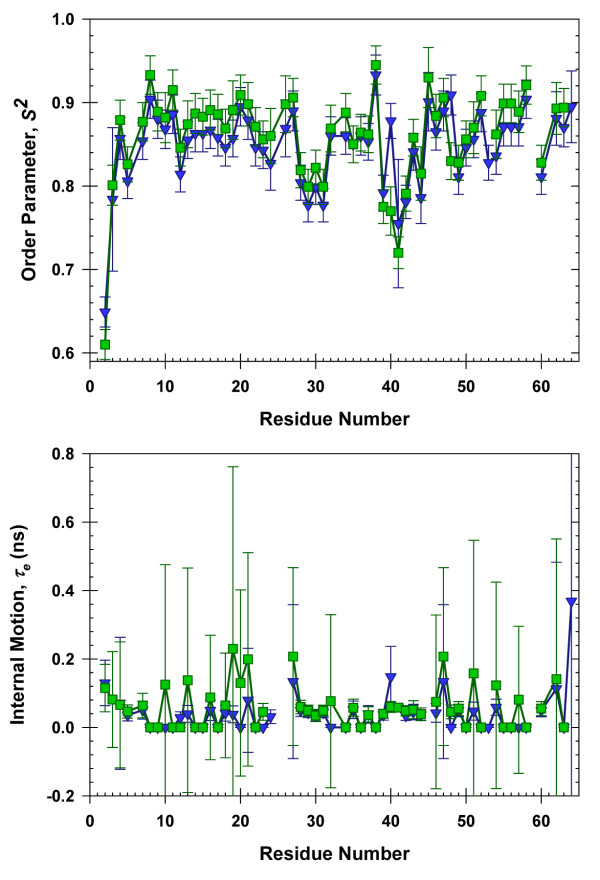
**CI2 dynamics in *p*-NIPAm-*co*-AAc**: Order parameters (upper panel) and timescales of internal motion (lower panel) for CI2 in dilute solution and in 10 g/L NIPAm-AAc.

Analysis of the *T*_*1*_, *T*_*2*_, and NOE data (Additional File 1) acquired in dilute solution and in 10 g/L *p*-NIPAm-*co*-AAc yielded the values for τ_m_, *S*^*2*^, and τ_e_. The value of τ_m _was the same (4.1 ns) in dilute solution and in 10 g/L *p*-NIPAm-*co*-AAc, and is consistent with the value obtained by Shaw *et al. *in dilute solution [9]. Histograms of *S*^*2 *^and τ_e _*versus *residue number are shown in Figure 2. Linear least squares analysis of a plot of *S*^*2 *^in dilute solution *versus S*^*2 *^in crowded solution gives a slope of 1.0 ± 0.1, a y-intercept of 0.1 ± 0.1 and an R^2 ^value of 0.80.

### Amide Proton Exchange and Stability

**Figure 3:**
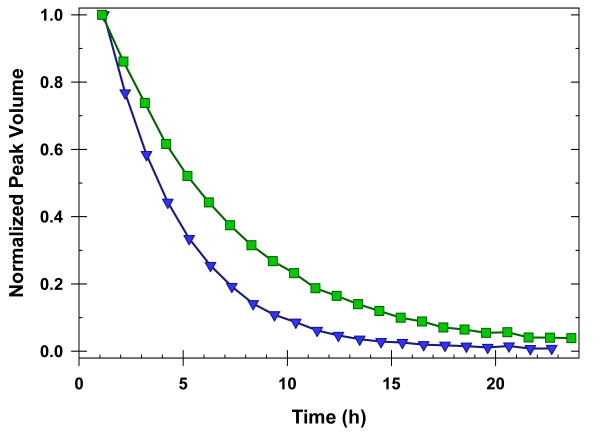
**Exchange Curves**: Amide proton exchange curves for Lys24 in dilute solution (blue triangles) and in 10 g/L *p*-NIPAm-*co*-AAc (green squares). Values for *k*_*obs *_are 7.53 ± 0.05 × 10^-5 ^± s^-1 ^in dilute solution and 4.55 ± 0.05 × 10^-5 ^s^-1 ^in 10 g/L *p*-NIPAm-*co*-AAc. These uncertainties are from non-linear least squares fitting and are smaller than the uncertainty from triplicate analysis.

**Figure 4:**
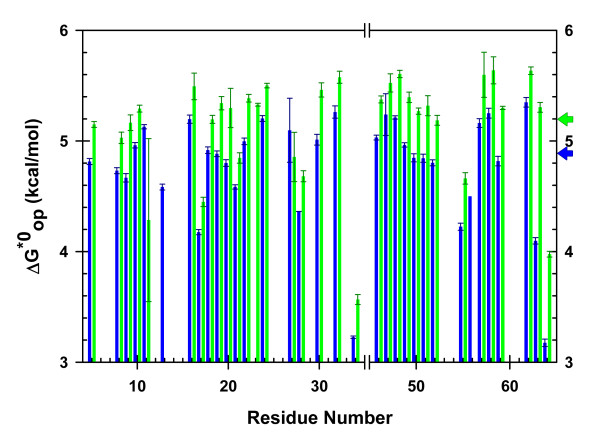
**CI2 stability in *p*-NIPAm-*co*-AAc**: Results are shown for dilute solution (blue) and 10 g/L *p*-NIPAm-*co*-AAc (green). Error bars reflect the standard error in *k*_*obs *_values from three trials. Colored arrows indicate the average *ΔG*^0*^_op _values for globally exchanging residues [20] in crowded (5.2 kcal/mol) and dilute (4.9 kcal/mol) solution.

Values for *k*_*obs *_were determined in triplicate for solutions in the presence and absence of 10 g/L *p*-NIPAm-*co*-AAc. Exchange was slowed in 10 g/L *p*-NIPAm-*co*-AAc compared to dilute solution (Figure 3). Values of *ΔG*^0*^_op _were determined by using values for *k*_*int *_calculated from SPHERE [14] and *k*_*obs *_values from amide proton exchange experiments. A listing of values is given in Additional File 2. A histogram of *ΔG*^0*^_op _*versus *residue number is shown in Figure 4.

## Discussion

The volume occupancy of *p*-NIPAm-*co*-AAc solutions defines the degree of crowding. Using a hydrodynamic radius of 312 nm and a molecular weight of 1 GDa, the microgel in a 10 g/L solution occupies ~70% of the solution volume at pH 5.4 and 37°C (the conditions used in our experiments). The practical limit of spherical packing is 64% volume occupancy [15], but soft materials such as microgels can be "overpacked" [16]. Our solutions, however, were still in the liquid state, meaning our value for volume occupancy is likely an overestimate. The high value does, however, suggest that experimental conditions were within the realm of crowding, as other systems show crowding effects at less than 20% volume occupancy [17,18].

Although the microgel slowed exchange (Figure 3), it was necessary to perform control experiments to ensure that stability values could be obtained under both sets of conditions. First, we confirmed that amide proton exchange from the open state (*k*_*int*_, Scheme 1) is rate limiting. Under this condition, pairs of proximal amide protons, A and B, open with the same frequency, but with different *k*_*int *_values. That is, amide proton exchanges for A and B are uncorrelated. By observing the decay of an amide-amide NOESY crosspeak corresponding to a resonance coupling between A and B, it is possible to determine whether their exchange is correlated or uncorrelated. If the exchange is uncorrelated, the decay curve of the amide-amide crosspeak should equal the product of the individual amide proton decay curves [19,20],

In this instance, the overall exchange rate of the amide-amide crosspeak will correspond to the sum of the individual exchange rate constants,

All these rates can be assessed from a series of ^15^N-filtered ^1^H-^1^H NOESY spectra acquired under exchange conditions [10].

As shown in Table 1, the exchange rates observed for the amide-amide crosspeaks for CI2 in both dilute solution and in 10 g/L *p*-NIPAm-*co*-AAc are, within the uncertainty of the experiment, the sums of their respective individual exchange rates, indicating that the exchanges are uncorrelated. We conclude that exchange from the open state is rate limiting, allowing determination of stability from amide proton exchange rates.

Second, we must determine if the microgel changes *k*_*int *_from the values determined in dilute solution. The dilute solution value for each residue is calculated by using the computer program, SPHERE [14] (http://www.fccc.edu/research/labs/roder/sphere/). The program uses values from the exchange of free peptides [21], and relies solely on the primary structure of the test protein. We assessed whether *k*_*int *_is affected by adding *p*-NIPAm-*co*-AAc by using the CLEANEX-PM experiment [13]. We measured the exchange rate of the His37 amide proton, which is fully exposed in the flexible loop region of CI2 (residues 35-44). The data indicated that the intrinsic rate of exchange in 10 g/L *p*-NIPAm-*co*-AAc (8 ± 2 s^-1^) is within uncertainty of the value in dilute solution (11 ± 2 s^-1^). These results suggest that *k*_*int *_values can be used without alteration. Having shown that it is valid to use *k*_*obs *_and *k*_*int *_values to obtain opening free energies, we constructed histograms of *ΔG*^0*^_op _values *versus *residue number (Figure 4).

### Dynamics and Stability

Crowding involves two different types of effects on protein stability: volume exclusion and chemical interactions. Volume exclusion is expected to stabilize protein native states, whereas chemical interactions can be stabilizing or destabilizing [6]. Attractive chemical interactions are expected to impede rotational dynamics, and the microgel used here is known to have favorable electrostatic interactions with proteins at low ionic strength [22]. Our data were collected at pH 5.4, where the microgel is negatively charged. The truncated form of CI2 we use has an isoelectric point (pI) of 6. Therefore, the polymer and CI2 are oppositely charged, and one might expect an attractive interaction.

Our observation that the order parameters (*S*^*2*^), the timescale of internal motion (*τ*_*e*_), and the rotational correlation time (*τ*_*m*_), are unchanged by the polymer indicates the absence of significant chemical interactions between the polymer and CI2. The lack of interaction probably arises because we used an ionic strength of 50 mM, which minimizes binding [22]. Therefore, we only consider contributions from volume exclusion effects.

The patterns of *ΔG*^0*^_op _values along the amino acid sequence (Figure 4) are the same in dilute solution as they are in the microgel solution, suggesting that the microgel does not alter the open states of CI2. The *ΔG*^0*^_op _values in the microgel are uniformly larger than the values for dilute solution, indicating the polymer stabilizes the protein with a maximal stability increase of approximately 0.4 kcal/mol. Averaging the *ΔG*^0*^_op _values from residues known to be implicated in global unfolding [20] show that the microgel increases the overall stability from 4.9 kcal/mol to 5.2 kcal/mol. We cannot state with certainty that the increased stability arises from the polymeric nature of the microgel because its crosslinked nature makes determination of a suitable monomer unit difficult.

**Figure 5:**
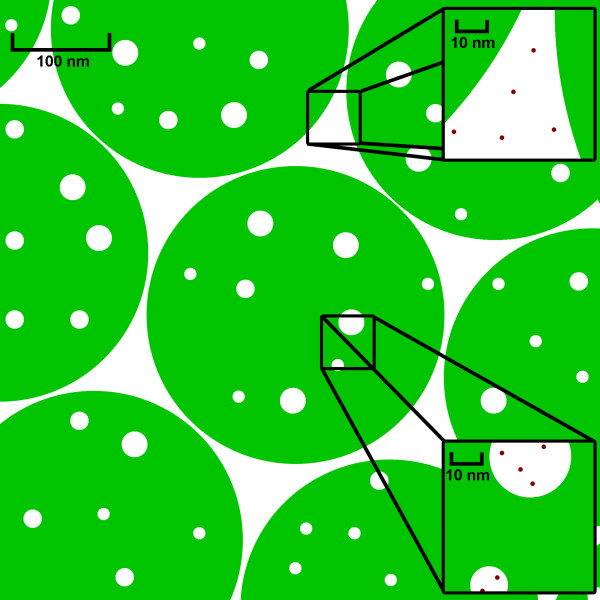
**Interstitial Spaces in *p*-NIPAm-*co*-AAc**: Depiction of the scale of microgel sizes for *p*-NIPAm-*co*-AAc (green) and CI2 (red). CI2 can exist in the spaces between crowder particles or within pores (of unknown size) without experiencing a change in environment compared to bulk water.

Considering the volume fraction estimate of ~70%, a 0.3 kcal/mol stability increase is quite small. A modest increase is anticipated, however, because the hydrodynamic radius of CI2 is only 1% that of the *p*-NIPAm-*co*-AAc microgels (Figure 5). In such a system, CI2 can occupy interstitial spaces between *p*-NIPAm-*co*-AAc microgels, putting CI2 in a dilute solution environment. Alternatively, the microgel particles probably have pores large enough to accommodate CI2 and water.

Next, we try to relate the stability change to the backbone dynamics data (Figure 2). The data indicate that the increased stability does not alter the ps-ns backbone dynamics. It has been proposed that stability changes are associated with alterations of ps-ns backbone dynamics [23,24]. Our results do not indicate a connection, because we observe increased stability without a change in ps-ns timescale dynamics. The most straightforward conclusion is that stability is not linked to backbone ps-ns dynamics. It is possible, however, that stability is reflected in slower (ms-s) motions [25].

## Conclusions

Even though the 10 g/L solution of *p*-NIPAm-*co*-AAc microgels occupy ~70% of solution volume, these conditions do not affect the ps-ns timescale backbone dynamics of CI2. The microgel, however, does have a modest stabilizing effect on the protein. These conclusions are explained by the fact that the majority of the protein occupies a water-like environment in interstitial spaces of the microgel particles. In the context of *p*-NIPAm-*co*-AAc as a drug delivery tool, this is promising information, supporting the notion that these microgels are biocompatible materials. It seems likely, however, that larger crowding agents such as *p*-NIPAm-*co*-AAc can have more noticeable effects when present in mixed solutions that also contain multiple sizes of crowders [26].

## Methods

^15^N-enriched CI2 was expressed and purified as described by Miklos *et al. *[10].

### Polymer Synthesis and Characterization

A general synthesis for NIPAm-AAc microgels is described by Jones and Lyon [27], but variations yield products with different properties (size, temperature, pH dependence, *etc.*) [28-30]. The microgels used here were prepared via aqueous, surfactant-free, free radical precipitation polymerization using 70 mM total monomer concentration. Briefly, *N*-isopropylacrylamide (0.6973 g) and *N,N'*-methylenebis(acrylamide) (0.0215 g) were dissolved in 99 mL of H2O and filtered through a 0.8 μm syringe filter into a round bottom flask. The mixture was bubbled with N_2 _(g) and heated to 70°C (± 2°C) over ~1 h. Acrylic acid (46 μL) was then added. Polymerization was initiated by adding a solution of (NH_4_)_2_S_2_O_8 _(0.0226 g) dissolved in 1 mL of H_2_O. This reaction was stirred at 70°C (± 2°C) under a blanket of N_2 _(g) for 4 h and was stirred and cooled overnight. The mixture was filtered through Whatman #2 paper and stored. Aliquots of the resultant colloidal dispersion were purified with centrifugation at 15,000 × g, decanted, and resuspended in H_2_O. This process was performed three times. The particles were then lyophilized to yield a white powder.

The microgels were characterized after suspension in sodium acetate (pH 5.4) and passage through a 0.8 μm filter. This solution was sonicated for 5 min, allowed to equilibrate for 30 min, then analyzed by using multi-angle laser light scattering (MALLS) [12].

### NMR

HSQC-detected and NOESY-HEX experiments were performed on a 500 MHz Varian Inova spectrometer equipped with a triple-resonance HCN cold probe as described by Miklos *et al. *[10]. CLEANEX-PM experiments were conducted as described by Hwang *et al. *[13] with a 600 MHz Varian Inova spectrometer equipped with a triple-resonance HCN probe with three-axis gradients system.

^15^N *T*_*1 *_and *T*_*2 *_relaxation times and ^15^N{^1^H} NOEs were measured as described by Kay *et al. *[31]. Experiments were performed on the 600 MHz spectrometer. Lipari-Szabo model free analysis [7] was performed with the software package Relaxn 2.2. [32]. The majority of residues were fit with the original model-free formalism [4] to yield τ_m_, *S*^*2 *^and τ_e_.

## Authors' contributions

ACM, CL, LAL, and GJP designed the research; ACM and CL performed the NMR experiments; CDS prepared and characterized microgels; ACM and GJP wrote the manuscript; All authors read and approved the final manuscript.

## Supplementary Material

Additional file 1 **^15^N *T*_*1*_, ^15^N *T*_*2*_, and ^1^H-^15^N NOEs for CI2**. A table containing ^15^N *T*_*1*_, ^15^N *T*_*2*_, and ^1^H-^15^N NOE values for CI2 in dilute solution and 10 g/L *p*-NIPAm-*co*-AAc at 37°C, pH 5.4. Click here for file

Additional file 2 **CI2 Stability Values**. A table containing *ΔG*^0*^_op _values and standard error from triplicate results for CI2 in 10 g/L *p*-NIPAm-*co*-AAc at 37°C, pH 5.4. Click here for file
