# Supplementary material for: An upper limit for macromolecular crowding effects
Source: BMC Biophys. 2011 May 31;4:13. doi: 10.1186/2046-1682-4-13 (PMC3120801; doi:10.1186/2046-1682-4-13)
Supplement: Additional file 1 — 15N T1, 15N T2, and 1H-15N NOEs for CI2. A table containing 15N T1, 15N T2, and 1H-15N NOE values for CI2 in dilute solution and 10 g/L p-NIPAm-co-AAc at 37°C, pH 5.4. [file 2046-1682-4-13-S1.DOC]

**Table S1:** ^15^N *T_1_*, ^15^N *T_2_*, and ^1^H-^15^N NOEs for CI2 in dilute solution and 10 g/L *p*­NIPAm-*co*-AAc at 37 °C, pH 5.4. Errors are uniform across residues (*T_1_*, ±0.02 s; *T_2_*, ±0.01 s; NOE, ±0.04).

|  | ***T_1_* (in s)** | | ***T_2_*(in s)** | | **NOE** | |
| --- | --- | --- | --- | --- | --- | --- |
| **Residue** | **Dilute** | **Crowded** | **Dilute** | **Crowded** | **Dilute** | **Crowded** |
| K2 | 0.53 | 0.58 | 0.21 | 0.20 | 0.31 | 0.26 |
| T3 | 0.44 | 0.48 | 0.18 | 0.16 | 0.65 | 0.64 |
| E4 | 0.44 | 0.46 | 0.16 | 0.14 | 0.70 | 0.73 |
| W5 | 0.47 | 0.49 | 0.17 | 0.15 | 0.70 | 0.70 |
| E7 | 0.46 | 0.47 | 0.16 | 0.14 | 0.70 | 0.71 |
| L8 | 0.43 | 0.44 | 0.16 | 0.14 | 0.77 | 0.76 |
| V9 | 0.43 | 0.46 | 0.16 | 0.14 | 0.75 | 0.74 |
| G10 | 0.44 | 0.45 | 0.16 | 0.14 | 0.73 | 0.73 |
| K11 | 0.44 | 0.46 | 0.16 | 0.14 | 0.74 | 0.77 |
| S12 | 0.46 | 0.48 | 0.17 | 0.15 | 0.72 | 0.76 |
| V13 | 0.44 | 0.46 | 0.17 | 0.15 | 0.72 | 0.71 |
| E14 | 0.45 | 0.46 | 0.16 | 0.14 | 0.77 | 0.74 |
| E15 | 0.45 | 0.47 | 0.16 | 0.14 | 0.76 | 0.78 |
| A16 | 0.45 | 0.46 | 0.16 | 0.14 | 0.71 | 0.72 |
| K17 | 0.45 | 0.46 | 0.16 | 0.14 | 0.74 | 0.75 |
| K18 | 0.45 | 0.47 | 0.16 | 0.14 | 0.71 | 0.72 |
| V19 | 0.45 | 0.44 | 0.16 | 0.14 | 0.72 | 0.73 |
| I20 | 0.44 | 0.45 | 0.15 | 0.14 | 0.73 | 0.73 |
| L21 | 0.44 | 0.45 | 0.17 | 0.14 | 0.70 | 0.70 |
| Q22 | 0.46 | 0.47 | 0.16 | 0.15 | 0.75 | 0.75 |
| D23 | 0.46 | 0.48 | 0.17 | 0.15 | 0.74 | 0.72 |
| K24 | 0.46 | 0.48 | 0.15 | 0.13 | 0.72 | 0.74 |
| E26 | 0.44 | 0.46 | 0.14 | 0.13 | 0.76 | 0.76 |
| A27 | 0.43 | 0.45 | 0.15 | 0.14 | 0.69 | 0.69 |
| Q28 | 0.48 | 0.49 | 0.17 | 0.15 | 0.67 | 0.67 |
| A29 | 0.47 | 0.49 | 0.18 | 0.16 | 0.67 | 0.68 |
| I30 | 0.47 | 0.49 | 0.18 | 0.15 | 0.70 | 0.72 |
| V31 | 0.47 | 0.49 | 0.18 | 0.16 | 0.67 | 0.68 |
| L32 | 0.43 | 0.45 | 0.17 | 0.15 | 0.73 | 0.73 |
|  |  |  |  |  |  |  |
|  |  |  |  |  |  |  |
|  |  |  |  |  |  |  |
|  |  |  |  |  |  |  |
|  |  |  |  |  |  |  |
|  |  |  |  |  |  |  |
|  |  |  |  |  |  |  |
|  |  |  |  |  |  |  |
|  |  |  |  |  |  |  |
|  | ***T_1_*(in s)** | | ***T_2_*(in s)** | | **NOE** | |
| **Residue** | **Dilute** | **Crowded** | **Dilute** | **Crowded** | **Dilute** | **Crowded** |
|  |  |  |  |  |  |  |
| V34 | 0.45 | 0.46 | 0.16 | 0.14 | 0.80 | 0.80 |
| G35 | 0.44 | 0.47 | 0.17 | 0.15 | 0.70 | 0.70 |
| T36 | 0.44 | 0.47 | 0.17 | 0.15 | 0.77 | 0.78 |
| H37 | 0.44 | 0.47 | 0.17 | 0.15 | 0.72 | 0.74 |
| V38 | 0.43 | 0.45 | 0.15 | 0.13 | 0.73 | 0.74 |
| T39 | 0.48 | 0.53 | 0.17 | 0.16 | 0.69 | 0.68 |
| M40 | 0.44 | 0.52 | 0.15 | 0.15 | 0.64 | 0.63 |
| Y42 | 0.49 | 0.51 | 0.18 | 0.16 | 0.69 | 0.67 |
| R43 | 0.46 | 0.49 | 0.16 | 0.14 | 0.70 | 0.71 |
| I44 | 0.48 | 0.50 | 0.08 | 0.08 | 0.69 | 0.71 |
| D45 | 0.43 | 0.44 | 0.12 | 0.12 | 0.73 | 0.74 |
| R46 | 0.44 | 0.46 | 0.16 | 0.14 | 0.72 | 0.74 |
| V47 | 0.43 | 0.45 | 0.15 | 0.14 | 0.69 | 0.69 |
| R48 | 0.41 | 0.47 | 0.16 | 0.16 | 0.74 | 0.71 |
| L49 | 0.45 | 0.47 | 0.18 | 0.16 | 0.68 | 0.69 |
| F50 | 0.45 | 0.47 | 0.17 | 0.15 | 0.77 | 0.76 |
| V51 | 0.44 | 0.45 | 0.17 | 0.15 | 0.71 | 0.71 |
| D52 | 0.44 | 0.46 | 0.16 | 0.14 | 0.74 | 0.74 |
| K53 | 0.44 | 0.47 | 0.18 | 0.16 | 0.76 | 0.75 |
| L54 | 0.45 | 0.46 | 0.17 | 0.15 | 0.68 | 0.70 |
| D55 | 0.44 | 0.46 | 0.16 | 0.14 | 0.75 | 0.75 |
| N56 | 0.44 | 0.46 | 0.16 | 0.14 | 0.75 | 0.75 |
| I57 | 0.45 | 0.46 | 0.16 | 0.14 | 0.73 | 0.73 |
| A58 | 0.42 | 0.44 | 0.16 | 0.14 | 0.74 | 0.76 |
| E59 | 0.47 | 0.49 | 0.19 | 0.17 | 0.73 | 0.74 |
| V60 | 0.45 | 0.47 | 0.18 | 0.16 | 0.68 | 0.69 |
| R62 | 0.43 | 0.45 | 0.16 | 0.14 | 0.72 | 0.74 |
| V63 | 0.45 | 0.46 | 0.16 | 0.14 | 0.75 | 0.74 |
| G64 | 0.41 | 0.43 | 0.15 | 0.14 | 0.71 | 0.71 |
