# Supplementary material for: An upper limit for macromolecular crowding effects
Source: BMC Biophys. 2011 May 31;4:13. doi: 10.1186/2046-1682-4-13 (PMC3120801; doi:10.1186/2046-1682-4-13)
Supplement: Additional file 2 — CI2 Stability Values. A table containing ΔG0*op values and standard error from triplicate results for CI2 in 10 g/L p-NIPAm-co-AAc at 37°C, pH 5.4. [file 2046-1682-4-13-S2.DOC]

**Table S2:** *ΔG^0*^_op_* values and standard error from triplicate results for CI2 in 10 g/L *p*­NIPAm-*co*-AAc at 37 °C, pH 5.4. *ΔG^0’^_op_* values for dilute solution conditions can be found in Miklos *et al.* [7].

| **Residue** | ***ΔG^0*^_op_*** | **Error** |  | **Residue** | ***ΔG^0*^_op_*** | **Error** |
| --- | --- | --- | --- | --- | --- | --- |
| W5 | 5.36 | 0.02 |  | L32 | 5.8 | 0.1 |
| L8 | 5.2 | 0.1* |  | V34 | 3.71 | 0.04 |
| V9 | 5.37 | 0.06 |  | R46 | 5.59 | 0.03 |
| G10 | 5.51 | 0.03 |  | V47 | 5.7 | 0.1 |
| K11 | 3.8 | 0.03* |  | R48 | 5.83 | 0.03 |
| A16 | 5.7 | 0.1 |  | L49 | 5.61 | 0.04 |
| K17 | 4.63 | 0.04 |  | F50 | 5.48 | 0.02 |
| K18 | 5.40 | 0.03 |  | V51 | 5.5 | 0.1 |
| V19 | 5.6 | 0.1 |  | D52 | 5.40 | 0.04 |
| I20 | 5.5 | 0.2 |  | D55 | 4.85 | 0.04 |
| L21 | 5.04 | 0.04 |  | I57 | 5.8 | 0.2 |
| Q22 | 5.60 | 0.03 |  | A58 | 5.9 | 0.1 |
| D23 | 5.54 | 0.01 |  | E59 | 5.51 | 0.01 |
| K24 | 5.72 | 0.02 |  | R62 | 5.86 | 0.03 |
| A27 | 5.05 | 0.2 |  | V63 | 5.52 | 0.04 |
| Q28 | 4.87 | 0.04 |  | G64 | 4.14 | 0.02 |
| I30 | 5.7 | 0.1 |  |  |  |  |

*Error represents range from duplicate results rather than standard error from triplicate results.
